# Supplementary material for: Low Core Losses of Fe-Based Soft Magnetic Composites with an Zn-O-Si Insulating Layer Obtained by Coupling Synergistic Photodecomposition
Source: Materials (Basel). 2022 Dec 5;15(23):8660. doi: 10.3390/ma15238660 (PMC9738613; doi:10.3390/ma15238660)
Supplement: Supplementary file 1 [file materials-15-08660-s001.zip › materials-2011052-supplementary.pdf]

Supplementary Material

Low core losses Fe-based soft magnetic composites with Zn-O-Si insulating layer obtained by coupling synergistic photodecomposition

Siyuan Wang, Jingwu Zheng*, Danni Zheng, Liang Qiao, Yao Ying, Yiping Tang, Wei Cai, Wangchang Li, Jing Yu, Juan Li, Shenglei Che*

Research Center of Magnetic and Electronic Materials, College of Materials Science and Engineering, Zhejiang University of Technology, Hangzhou 310014, China

Corresponding author. E-mail address: zhengjw@zjut.edu.cn (Jingwu Zheng); cheshenglei@zjut.edu.cn (Shenglei Che)

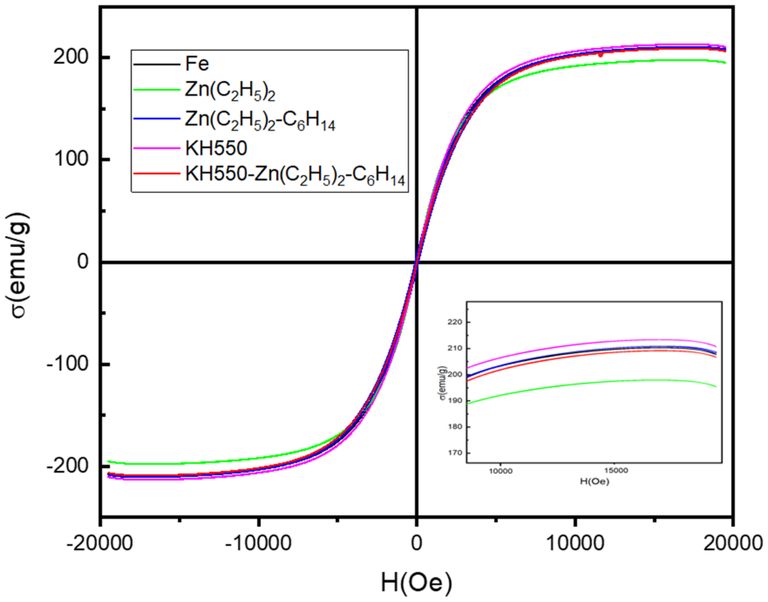

Figure S1. Magnetisation curves of iron powder and photolytically coated iron powder with different treatments.

Figure S1 shows the magnetization curve of iron powder and different photolytically coated iron powder with different treatments. It can be seen that the coercivity of these samples has only a small change, indicating that the treatment method of photodecomposition coating has little effect on the H_c of iron powder.

It can be seen that only the saturated magnetization (Ms) of iron powder coated by photodecomposition of diethyl zinc solution not diluted by n-hexane decreases, while that of other groups only changes slightly (The small changes mentioned above are due to the inevitable errors caused by the test). Combined with XRF, it can be concluded that the addition of diethylzinc photodegradation coating will introduce more non-magnetic phase Zn or ZnO, leading to the decrease of Ms.

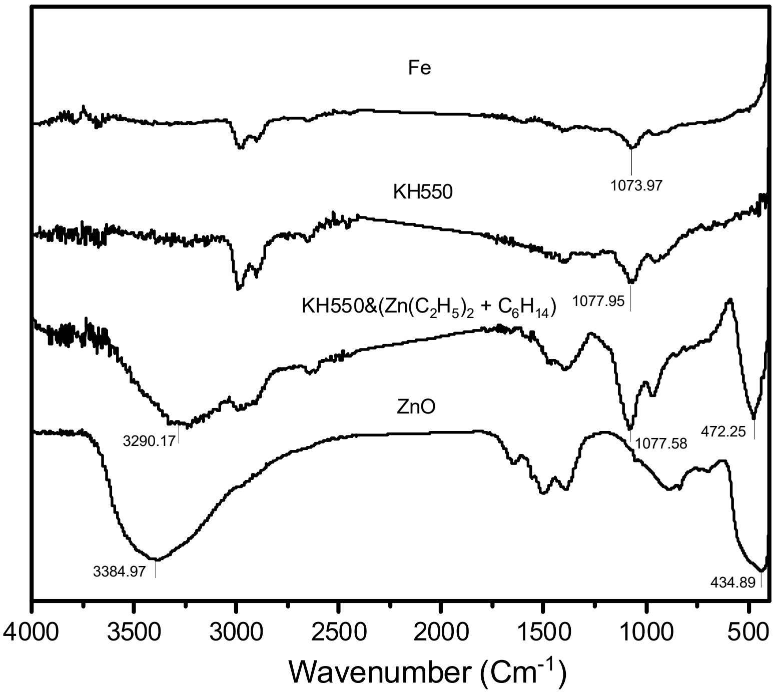

Figure S2. IR of iron powder; iron powder surface treated with 5 wt.% KH550; iron powder surface treated with 5 wt.% KH550&(Zn (C_2H_5) $_2$+C_6H_{14}) and ZnO obtained by photodecomposition.

In the Figure S2, 434.89cm^{-1} is the characteristic peak formed by Zn-O bond vibration, 1077.95cm^{-1} is the characteristic peak formed by the stretching vibration of Si-O bond, 3384.97cm^{-1} is the characteristic peak of -OH stretching vibration. It can be seen from the figure that the raw iron powder also shows an absorption peak at 1073.97cm^{-1}. It may be that the purchased raw iron powder is also subject to coupling treatment in advance to prevent oxidation, but the strength of the stretching vibration peak of the Si-O bond at 1077.95cm^{-1} of the iron powder after the re-coupling treatment in this experiment has significantly increased. The ZnO formed by photodecomposition has a characteristic peak formed by the vibration of Zn-O bond at 434.89cm-1. However, the characteristic absorption peaks of Si-O bond and Zn-O bond appear at 1077.58cm^{-1} and 472.25cm^{-1} respectively for the iron powder coated with ZnO after pre-coupling and photodecomposition. The position of the characteristic peak of Zn-O moves from 434.89cm^{-1} to 472.25cm^{-1}, the intensity of

-OH stretching vibration peak appeared at 3290.17 cm^{-1} is slightly lower than that of -OH stretching vibration peak of ZnO sample. It can be inferred from this that after pre-coupling and photodecomposition coating ZnO of iron powder, the surface will adsorb and form a chemical bond similar to Zn-O-Si.

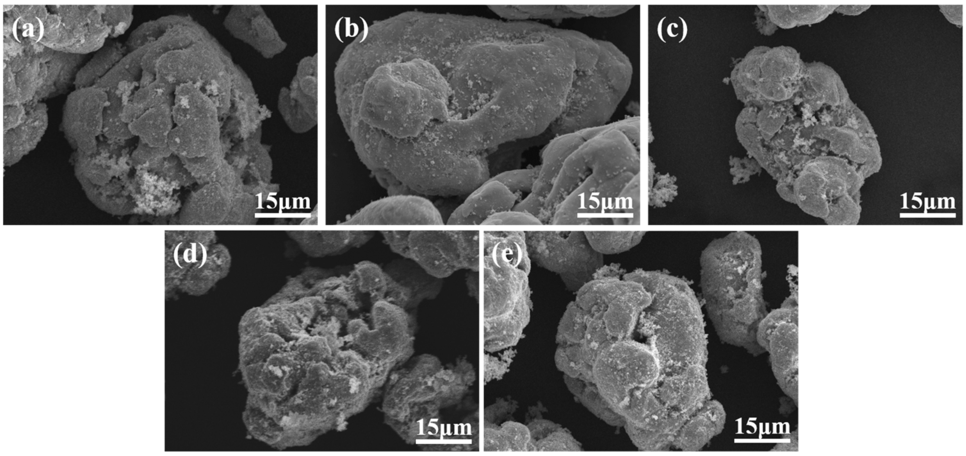

Figure S3. SEM morphology of iron powders treated with different KH550 ratios (a) 2 wt.%, (b) 3 wt.%, (c) 4 wt.%, (d) 5 wt.% and (e) 6 wt.% after photolytic coating

In this paper, we used 5 wt.% KH550 of the weight of magnetic powder for pre-coupling. The amount of coupling agent is the result of our optimization. The specific optimization results are shown in Figures S3 and S4.

Figure S3 shows the SEM morphology of the powder obtained after the iron powder was treated with KH550 in different proportions and coated through photodecomposition. It can be observed that when the treatment proportion is low, there are fewer Zn-O-Si compounds on the surface of the powder. When the KH550 ratio rises to 4 wt.%, the sample surface is basically covered by insulating particles, but there are also some irregular places where no photodecomposition products are deposited. Figure S3 When the KH550 ratio is further increased, it can be seen that the irregular parts of iron powder such as pits are also deposited with photodecomposition products. At this time, the iron powder is completely covered by the insulating compound, and a complete insulating layer is formed on the surface.

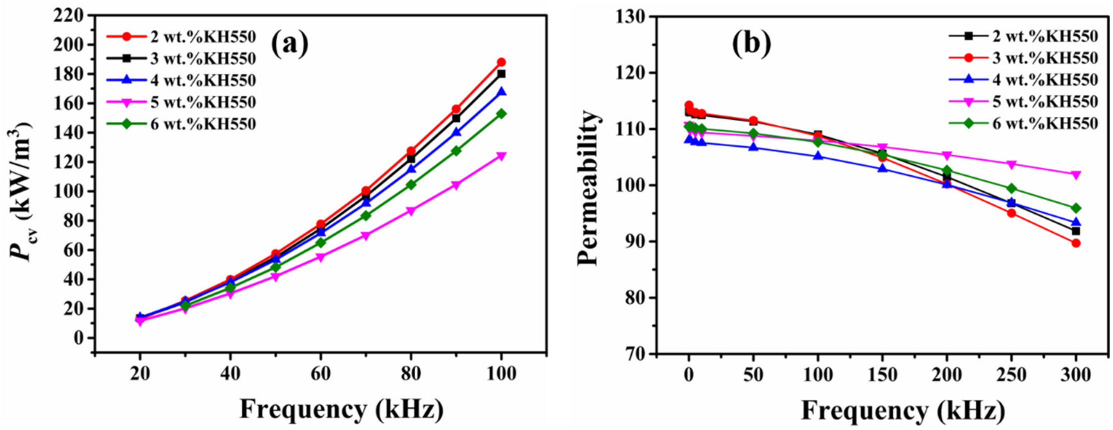

Figure S4. (a) Loss and (b) effective permeability of the SMC rings obtained after coating with iron powder treated with 2 wt.%, 3 wt.%, 4 wt.%, 5 wt.% and 6 wt.% KH550

Figure S4 shows the magnetic loss and effective permeability of the magnetic ring made of iron powder after being treated with KH550 at different proportions and then coated by photodecomposition. When the KH550 ratio increases, the magnetic loss first decreases and then increases. It is speculated that when the KH550 increases, more insulating layers can be deposited, making the insulating layer denser, so the eddy current loss decreases and the total loss decreases. When too many insulating products are deposited, too much nano Zn-O-Si is introduced, which increases the domain wall and the specific surface area of the sample, leads to the increase of hysteresis loss, so the total loss rises again. The permeability of the samples decreased with the KH550 treatment ratio. When the treatment ratio was 5 wt.%, the frequency stability of the permeability was good. Therefore, when the KH550 ratio is 5 wt.%, the overall performance of the sample is the best.
